# Constant neuropilar ratio in the insect brain

**DOI:** 10.1038/s41598-020-78599-2

**Published:** 2020-12-08

**Authors:** Alexey A. Polilov, Anastasia A. Makarova

**Affiliations:** Department of Entomology, Biological Faculty, Lomonosov Moscow State University, 119234 Moscow, Russia

**Keywords:** Entomology, Zoology

## Abstract

Revealing scaling rules is necessary for understanding the morphology, physiology and evolution of living systems. Studies of animal brains have revealed both general patterns, such as Haller's rule, and patterns specific for certain animal taxa. However, large-scale studies aimed at studying the ratio of the entire neuropil and the cell body rind in the insect brain have never been performed. Here we performed morphometric study of the adult brain in 37 insect species of 26 families and ten orders, ranging in volume from the smallest to the largest by a factor of more than 4,000,000, and show that all studied insects display a similar ratio of the volume of the neuropil to the cell body rind, 3:2. Allometric analysis for all insects shows that the ratio of the volume of the neuropil to the volume of the brain changes strictly isometrically. Analyses within particular taxa, size groups, and metamorphosis types also reveal no significant differences in the relative volume of the neuropil; isometry is observed in all cases. Thus, we establish a new scaling rule, according to which the relative volume of the entire neuropil in insect brain averages 60% and remains constant.

## Introduction

Large-scale studies of animal proportions supposedly started with the publication D'Arcy Wentworth Thompson's book *Growth and Forms*^1^. In fact, the first studies on the subject appeared long before the book (e.g.^2^), but it was Thomson's work that laid the foundations for this discipline, which, following the studies of Julian Huxley^3,4^, became a major fundamental and applied area of science^5–8^. Allometry and scaling of living systems are being studies within that area to this day. Studies of brain allometry are important for understanding the functional principles and evolution of animal nervous systems^9–11^. They have revealed both general patterns, such as Haller's rule, according to which the relative size of the brain decreases with decreasing body^12^, and patterns that hold true only for particular groups of animals.

Certain patterns of evolutionary and static allometry of the insect nervous system have been shown both for the entire central nervous system and brain and for particular synapse-rich neuropils of the brain. Increasing relative size with decreasing body size (according to Haller's rule) has been shown both for the brains of insects^12–26^ and for their entire central nervous systems^12,16,18,25,27,28^. Exceptions to this rule include particular lines in cultures of *Trichogramma*^29^ and *Nasonia*^30^. The sizes of particular synapse-rich neuropils of the brain can differ considerably between different insects and even within one species; they depend on many factors, such as the body size^14,17,20–23^, caste^26,31–41^, sex^35,42–48^, sociality^49,50^, ecology^24,45,46,51^, circadian rhythm type^52^, migratory activity^46,53^, and even age of the individual^17,39^. Ontogenetic allometry of the central nervous system, the brain, and synapse-rich neuropils has been described in insects with different types of development^54–57^ ^and others^.

The ganglia and brain of arthropods have the same general organization and consist of neuropil formed by the processes of cells and of the cell body rind (cortex) formed by the bodies of these cells^58^. There is not much data on the total volume of the neuropil of the brain in insects, since in the majority of studies only volumes or relative volumes for a few brain regions are reported. For the few data available, the ratio of neuropil volume to cell body rind volume are similar across insects^20–23^, but no large-scale analysis of this ratio was performed. The purpose of this study is to analyze the ratio and allometry of the neuropil and the cell body rind in the brains of a wide range of insects.

## Results and discussion

**Figure 1:**
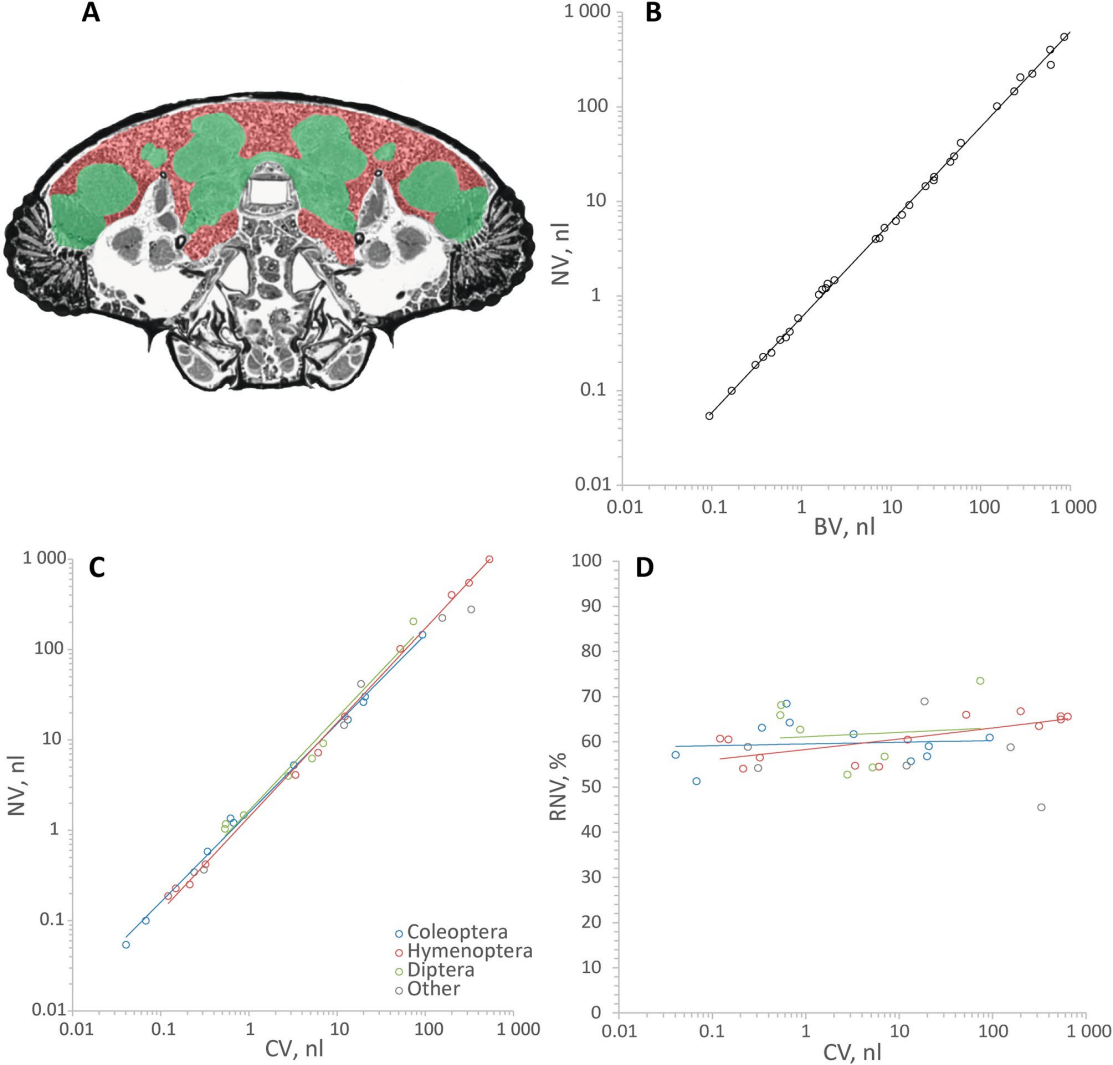
Scaling of neuropil in insect brains. (**A**) neuropil (green) and cell body rind (red) on a histological cross section of head of featherwing beetles *Acrotrichis grandicollis* (Coleoptera: Ptiliidae); (**B**) dependence of neuropil volume (NV) on brain volume (BV) in insects in general; (**C**) dependence of NV on cell body rind volume (CV) in major insect orders; (**D**) dependence of relative neuropil volume (RNV) on CV in major insect orders. All scales are logarithmic, except Y-axis in (**D**). For results of allometric analysis, see Table 2.

Analysis of our data and all available published data (Table 1) showed that adult insects generally have the same ratio of the total neuropil volume (NV) to brain volume (RNV) and it averages 60.5% ± 5.7. Allometric analysis for insects in general showed that the volume of the neuropil changes isometrically (the slope of the ratio of the NV to the volume of the brain (BV) or to the cell body rind volume (CV) does not differ significantly from 1; Table 2, Fig. 1). Exploratory analysis of particular taxonomic groups, size groups, and types of metamorphosis, based on samples of limited sizes, also revealed isometry and showed no significant differences between groups in RNV, slope or elevation (Table 2).

**Table 1:** Brain (BV) and neuropil volumes (NV) in insects (in cases of several measurements for one species, mean ± SD (n) are given).

Order	Species	BV, nL	NV, nL	RNV, %	Source of data (if taken from literature)
Zygentoma	*Lepisma saccharina*	60.25	41.52	68.9	
Orthoptera	*Acheta domesticus*	378.9 ± 123.2 (5)	222.7	58.8 ± 1.6	^56^
Blattoptera	*Periplaneta americana*	782.1 (6)	436.6	55.8	^84^
Psocoptera	*Copostigma* sp.	24.32	14.49	59.6	
Psocoptera	*Liposcelis bostrychophila*	0.583	0.343	58.9	
Thysanoptera	*Heliothrips haemorrhoidalis*	0.674	0.365	54.2	
Hemiptera	*Oncopeltus fasciatus*	–	–	53.0	^85^
Coleoptera	*Tetraphalerus bruchi*	46.12	26.19	56.8	
Coleoptera	*Ochthebius* sp.	8.49	5.24	61.7	
Coleoptera	*Acrotrichis grandicollis*	1.88	1.21	64.2	
Coleoptera	*Mikado* sp*.*	0.167	0.102	59.5	
Coleoptera	*Nanosella* sp*.*	0.094	0.054	57.1	
Coleoptera	*Aleochara* sp.	30.02	16.71	55.7	
Coleoptera	*Staphylinus caesareus*	238.8	145.5	60.9	
Coleoptera	*Atheta* sp.	0.921	0.581	63.1	
Coleoptera	*Semiadalia notata*	50.78	29.96	59.0	
Coleoptera	*Sericoderus lateralis*	1.97	1.35	68.4	
Hymenoptera	*Macroxyela ferruginea*	153.3	101.2	65.9	
Hymenoptera	*Anagrus* sp.	0.742	0.419	56.5	
Hymenoptera	*Anaphes flavipes*	0.376	0.228	60.5	
Hymenoptera	*Trichogramma evanescens*	0.308	0.187	60.7	
Hymenoptera	*Trichogramma telengai*	0.465	0.251	54.0	
Hymenoptera	*Hemiptarsenus* sp.	7.47	4.08	54.7	
Hymenoptera	*Nasonia vitripennis* (large)	30.4 ± 2.4 (17)	18.1 ± 1.6	60.4 ± 3.0	^30^
Hymenoptera	*Nasonia vitripennis* (small)	13.2 ± 1.7 (11)	7.2 ± 1.0	54.5 ± 2.9	^30^
Hymenoptera	*Apis florea*	600 ± 50 (8)	400	66.7	^59^
Hymenoptera	*Apis cerana*	860 ± 30 (8)	546	63.5	^59^
Hymenoptera	*Apis mellifera*	1 530 ± 80 (7)	993	64.9	^59^
Hymenoptera	*Apis dorsata*	1 560 ± 60 (8)	1 024	65.6	^59^
Hymenoptera	*Bombus impatiens*	1 850.0 ± 400 (25)	1 213	65.6	^59^
Lepidoptera	*Antheraea pernyi*	609.2 ± 48.2 (5)	276.7 ± 18.0	45.5	^55^
Diptera	*Mayetiola destructor*	1.72	1.17	68.1	
Diptera	*Culex pipiens*	16.1 ± 1.3 (10)	9.13 ± 0.7	56.7	^57^
Diptera	*Hydrellia albolabris*	6.79	3.99	52.7	
Diptera	*Corynoneura scutellata*	1.57	1.03	65.9	
Diptera	*Leptocera* sp.	2.34	1.47	62.7	
Diptera	*Drosophila melanogaster*	11.40 ± 0.7 (10)	6.19 ± 0.5	54.3	^57^
Diptera	*Musca domestica*	278.3	204.6	73.5	^86^

**Table 2:** Comparison of neuropil volumes in different groups of insects.

Sample	RNV	NV on BV			NV on CV			n
		Slope	Elevation	R^2^	Slope	Elevation	R^2^	
All insects	60.4 ± 5.7	1.006	− 0.227	0.999	1.017	0.176	0.993	37
Bogy length < 2 mm	59.0 ± 5.3	1.008	− 0.223	0.997	1.017	0.184	0.980	15
Body length ≥ 2 mm	60.5 ± 5.9	1.013	− 0.243	0.997	1.031	0.149	0.982	22
Coleoptera	59.8 ± 4.9	0.996	− 0.215	0.999	0.988	0.193	0.996	10
Hymenoptera	61.0 ± 4.8	1.017	− 0.242	0.999	1.045	0.146	0.998	12
Diptera	62.0 ± 7.7	1.014	− 0.216	0.997	1.044	0.213	0.974	7
Hemimetabolous	57.7 ± 7.9	0.992	− 0.261	0.998	1.034	0.139	0.993	7
Holometabolous	60.9 ± 5.5	1.009	− 0.225	0.999	1.015	0.182	0.993	30
Social bees	65.7 ± 1.2*	0.996	− 0.181**	0.998	0.984	0.315***	0.988	5
Non-social insects	59.1 ± 5.9*	1.001	− 0.224**	0.999	0.999	0.175***	0.990	32

Relative neuropil volume to brain volume (RNV, Mean % ± SD). Slope, elevation, and R^2^ from SMA allometric analysis of dependence of neuropil volume (NV) on brain volume (BV) and cell body rind volume (CV) (log); n is the number of species in sample; * RNV significantly different between sample(ANOVA p = 0.033); ** and *** significantly different elevations (elev.com p = 0.015 and 0.019, respectively).

The only exception is the social bees, in which the average values of RNV and elevation in allometric analysis are significantly higher than in other insects (Table 2). However, these data need to be verified, because all data on social insects are taken from a single study, and they differ from data obtained earlier; for instance, for the honeybee (*Apis mellifera*) RNV is 64.9% in that latest study^59^ and 61.3% in an earlier study^31^. The small sample size also does not allow making final conclusions about the supposedly unusual RNV of bees. Among all the RNV values, there is one that is somewhat out of the general sample: 45% for the moth *Antheraea pernyi*^55^, but these data were obtained long ago and it is possible that using modern methods, especially 3D modeling, will correct these measurements. It is also possible that this is an interesting exception from the general rule, but lack of data on other lepidopterans makes it impossible to discuss this at present. A very low RNV value has been reported in the drone of *Apis mellifera*: 46.6%^31^, and this phenomenon requires further study. Interestingly, the eyeless mutant of *Drosophila*, in which the brain is almost two thirds as large as in the wild type, retains the same RNV as in the wild type^57^. It was repeatedly shown previously that different methods of sample preparation can change the size of structures, including the brain^60^, which can introduce significant variance in morphometric data. Apparently, the neuropil and body rind have similar deformation parameters in cases of different sample preparation methods, since our analysis of the data obtained by different methods shows no large deviations.

In the parasitic wasp *Nasonia vitripennis* (Hymenoptera: Pteromalidae) RNV in a sample consisting of the largest individuals is higher than in a sample consisting of the smallest individuals of the same species and averages 60.4 and 54.5%, respectively^30^. But artificial selection of individuals from opposite extremes of the body size range (as used in that study), especially for parasitoids kept in a culture, in which the characteristics of the host and population density of the parasitoid strongly affect the body size of the latter^61,62^, considerably expanding the reaction norm compared to natural populations^63,64^, produces data that could be difficult to compare with those obtained from natural populations. There are some known examples of artificial selection affecting the allometry characteristics of structures, but when artificial selection stops, allometry returns to its initial state^65^.

It is especially interesting that the same RNV is retained in miniature insects, which often exhibit considerable changes in the structure of the brain: asymmetry, displacement into other segments, huge relative volume, multiple reduction in the number of neurons and their sizes^20–23,66^. A significant decrease in the size of the cell bodies of neurons in microinsects compared with larger representatives of related groups of insects leads to changes in the nuclear-cytoplasmic ratio, a decrease in the number and size of organelles in the cell, and an increase in the level of chromatin compaction^20,66,67^. We showed earlier that it is the size of the cell bodies of neurons, limited by the minimum size of the nucleus, that limits the miniaturization of the central nervous system, which in turn is the most important factor limiting the minimum body size of insects^67^. It could be assumed that a neuropil consisting of processes of cells with a small number of organelles could tolerate miniaturization better than the cell body rind and could reduce its relative volume in miniature forms. But this is not the case: even the smallest insects have the same RNV as large insects. This is probably due to the fact that the efficiency of neurons depends on the diameter of their processes. As calculated earlier, the noise effects of ion channels make it impossible to transmit impulses along axons with a diameter of less than 80 nm^68^, and these physical limitations probably limit the decrease in the neuropil volume.

A special place is occupied by the parasitic wasp *Megaphragma* (Hymenoptera: Trichogrammatidae), in which about 98% of the brain volume is occupied by the neuropil, due to the fact that the central nervous system of the adult is almost anucleate in all studied species of this genus^69–71^. Because of these fundamental differences in brain organization, *Megaphragma* was excluded from our analysis in this study.

The same ratio of the neuropil and the cell body rind that we describe for the insect brain is also found in measurements of the total central nervous system of insects and other arthropods. The relative neuropil volume of the entire central nervous system (RNVcns) and of particular thoracic ganglia separately for the parasitic wasp *Trichogramma telengai* is no different from RNV, and only the abdominal ganglia have a slightly lower relative neuropil volume^72^. In the moth *Antheraea pernyi*, the relative volume of the neuropil of the mesothoracic ganglion is 65%, and that of abdominal ganglion 4 is 53%^55^. In the house cricket (*Acheta domesticus*), the relative volume of neuropil is 66–73% in the thoracic ganglia, and 63% in the last abdominal ganglion^56^. In the collembolan *Orchesella villosa*, by the age of the start of breeding, RNV is about 70%, but with subsequent molts it can reach 84% by the time of death^73^. In the spider *Eratigena atrica*, the relative volume of the neuropil is 61.1%^74^. In the spider *Argiope aurantia* it is 71.3%^75^. Unfortunately, at present there is not enough data for a comprehensive analysis of RNV and RNVcns in arthropods in general, but it is possible that a large-scale study will eventually reveal common patterns.

Interestingly, although RNV remains constant, the relative volumes of particular synapse-rich neuropils of the brain can vary considerably between different insect species or even within the same species, and the sizes of particular synapse-rich neuropils depend on many factors (for review, see “Introduction”). Furthermore, an increase in the relative sizes of the synapse-rich neuropils of one modality occurs at the expense of a decrease in the sizes of neuropils of other modalities or the size of undifferentiated neuropil^46,51,52,76^ ^and^ ^others^. It is probably due to such compensations that RNV remains constant.

The structural plans of the brains of insects and vertebrates are fundamentally different and it is difficult to make direct comparisons. However, interestingly, the “wire fraction” (percentage of axons and dendrites) in different parts of the mouse brain is 3/5, and this is consistent with mathematical calculations of wiring optimization^77^. At the same time, the percentage of the cerebral cortex, which is occupied by the neuropil in humans and chimpanzees, differ considerably between different regions of the brain, 63–71% in chimpanzees and 77–84% in humans^78^. There are also a number of studies in which the volumes of the white and gray matters are evaluated. The relative volume of the gray matter decreases significantly with increasing body size and increasing number of neurons, and slopes and elevations differ between groups^79–81^. The relative volume of the gray matter in vertebrates varies between species within a very wide range, from 93% in the mouse *Mus musculus* to 66% in humans and to 50% in the elephant *Loxodonta africana*^81^. Thus, it can be assumed that the vertebrate brain shows a fundamentally higher diversity in the ratio of the neuropil to cellular regions than the insect brain. However, there is still not enough data for a large-scale analysis of different groups of animals.

## Conclusion

Thus, our large-scale analysis reveals a new scaling rule, according to which the ratio of the neuropil to the cell body rind of the brain of adult insects is the same (3:2) and the relative volume of the entire neuropil is constant and averages 60% of the brain volume.

## Methods

To analyze the relative volumes of the neuropil of the brain (RNV, the ratio of the total volume of the neuropil to the volume of the brain), 3D reconstructions of the brain made in the Bitplane Imaris program based on a series of histological sections were used. For these sections, the material was fixed in FAE (formaldehyde, acetic acid, and ethanol) and embedded in Araldite. The resulting blocks were used to make complete series sections 0.5–2 µm thick with a Leica RM2255 microtome. For 3D computer modeling, the series of sections were photographed under a Motic BA410 microscope. After, followed by the alignment of the resulting stack with FEI Amira. All structures were outlined manually and automatically recalculated as three-dimensional withusing Bitplane Imaris. The volumes of the brain and neuropil wereas calculated using 3D reconstructions in the Bitplane Imaris statistical module. The detailed methodology for processing the material and obtaining volumetric data has been described earlier^22,25,66^. The data on the adults of 24 species based on our original models are analyzed and published data are used for 13 other species (Table 2). A total of 37 species of 26 families and ten orders are analyzed, ranging in sizes from the smallest to the largest by a factor of over 4,000,000 by body volume and by a factor of 20,000 by brain volume. We used the classical definition of the brain as the supraesophageal ganglion (= supraesophageal zone^82^). Data analysis was performed in R using ANOVA to compare average values for samples and the smatr package^83^ for allometric analysis, using the standardized major axis (SMA). All analyzes were performed for all insects and in all four groups of samples (size group, orders, type of development, and sociality); in the groups of samples the values were compared between samples within the group (Table 2).
